# PD-L1 and Tumor Infiltrating Lymphocytes as Prognostic Markers in Resected NSCLC

**DOI:** 10.1371/journal.pone.0153954

**Published:** 2016-04-22

**Authors:** Malaka Ameratunga, Khashayar Asadi, Xihui Lin, Marzena Walkiewicz, Carmel Murone, Simon Knight, Paul Mitchell, Paul Boutros, Thomas John

**Affiliations:** 1 Department of Medical Oncology, Austin Health, Olivia-Newton John Cancer and Wellness Centre, Victoria, Australia; 2 Department of Pathology, Austin Health, Victoria, Australia; 3 Informatics & Biocomputing Program, Ontario Institute for Cancer Research, Toronto, Canada; 4 The Olivia Newton-John Cancer Research Institute, Victoria, Australia; 5 Department of Thoracics, Austin Health, Victoria, Australia; 6 Department of Medical Biophysics, University of Toronto, Toronto, Canada; 7 Department of Pharmacology & Toxicology, University of Toronto, Toronto, Canada; 8 School of Cancer Medicine, La Trobe University, Victoria, Australia; 9 University of Melbourne, Victoria, Australia; University of Algarve, PORTUGAL

## Abstract

**Introduction:**

Immune checkpoint inhibition has shifted treatment paradigms in non-small cell lung cancer (NSCLC). Conflicting results have been reported regarding the immune infiltrate and programmed death-ligand 1 (PD-L1) as a prognostic marker. We correlated the immune infiltrate and PD-L1 expression with clinicopathologic characteristics in a cohort of resected NSCLC.

**Methods:**

A tissue microarray was constructed using triplicate cores from consecutive resected NSCLC. Immunohistochemistry was performed for CD8, FOXP3 and PD-L1. Strong PD-L1 expression was predefined as greater than 50% tumor cell positivity. Matched nodal samples were assessed for concordance of PD-L1 expression.

**Results:**

Of 522 patients, 346 were node-negative (N0), 72 N1 and 109 N2; 265 were adenocarcinomas (AC), 182 squamous cell cancers (SCC) and 75 other. Strong PD-L1 expression was found in 24% cases. In the overall cohort, PD-L1 expression was not associated with survival. In patients with N2 disease, strong PD-L1 expression was associated with significantly improved disease-free (DFS) and overall survival (OS) in multivariate analysis (HR 0.49, 95%CI 0.36–0.94, p = 0.031; HR 0.46, 95%CI 0.26–0.80, p = 0.006). In this resected cohort only 5% harboured EGFR mutations, whereas 19% harboured KRAS and 23% other. KRAS mutated tumors were more likely to highly express PD-L1 compared to EGFR (22% vs 3%). A stromal CD8 infiltrate was associated with significantly improved DFS in SCC (HR 0.70, 95%CI 0.50–0.97, p = 0.034), but not AC, whereas FOXP3 was not prognostic. Matched nodal specimens (N = 53) were highly concordant for PD-L1 expression (89%).

**Conclusion:**

PD-L1 expression was not prognostic in the overall cohort. PD-L1 expression in primary tumor and matched nodal specimens were highly concordant. The observed survival benefit in N2 disease requires confirmation.

## Introduction

Recent years have seen a cascade of novel immunological agents with proven efficacy in multiple malignancies.[1–5] In particular, immune checkpoint-inhibitors targeting the interactions between cytotoxic T-lymphocyte associated protein (CTLA-4) and B7[4] and programmed cell death 1 (PD-1) and its ligand, programmed death-ligand 1 (PD-L1)[3] have shown unprecedented activity. Nevertheless, there is a significant knowledge deficit regarding the immune microenvironment, its role in the natural history of various tumors and its role in modulating response to these therapeutics.

Given that PD-L1 is inducible and may reflect homeostatic responses to immune activation [6], the immune infiltrate is important in contextualizing PD-L1 tumoral expression. The immune cell most consistently reported as prognostic has been CD8 expression [7–10], a marker for cytotoxic lymphocytes. Other markers evaluated in a range of tumors included CD3 (pan-lymphocyte marker), CD4 (helper T-lymphocyte marker), CD45 (marker for memory T- lymphocytes) and FOXP3 (a nuclear marker to delineate regulatory T-lymphocytes).[11–13] Additionally, cytokines previously suggested as prognostic include tumor IL-12RB2 and tumor IL-7R.[14] CD8 expression has generally been correlated with improved prognosis in solid malignancies, CD4 expression has largely been correlated with CD8 expression, and FOXP3 expression has been associated with no difference in outcomes or poorer prognosis. Nevertheless, there is substantial heterogeneity in the methodology and reproducibility of these findings.[7, 8, 11, 12, 15–26] There is also substantial debate regarding the spatial locations of the immune infiltrates and its correlate with survival with some authors suggesting stromal infiltrates being more prognostic, others suggesting tumoral infiltrates and more recently data suggesting the immune interface at the invasive margin as being most prognostic. [7, 18, 27]

To clarify the influence of PD-L1 expression and the stromal CD8+ lymphocyte infiltrate as prognostic factors in this patient cohort, we comprehensively profiled the immune microenvironment in a large retrospective cohort of resected NSCLC using a tissue microarray (TMA). Furthermore, we sought to clarify whether PD-L1 status was maintained in matched nodal samples from a subset of patients with tissue available from locoregionally advanced disease

## Materials and Methods

### Study design and patients

Consecutive patients undergoing curative surgery for NSCLC during the period from 1992 to 2010 at the Austin Hospital, Victoria, Australia were captured in a prospectively maintained database. Clinicopathologic data were reviewed and collated. The revised 2010 AJCC staging manual was used for classification of stage.[28] The project was approved by the Austin Health Human Research Ethics Committee. This was a retrospective cohort analysis and individual consent was waived on the provision that the cohort was de-identified and that no identifying features were included. The reporting of clinical data and biomarker expression was conducted in accordance with the REMARK guidelines.[29]

### NSCLC Tissue Microarray (TMA)

Lung cancer specimens were histologically reviewed by an experienced pathologist. Representative areas of tumor tissue were selected and marked, prior to being sampled for the TMA. One millimeter cores of formalin fixed paraffin embedded (FFPE) tissues in triplicate from a consecutive series of surgically resected lobectomy and pneumonectomy specimens were used for the TMA.

### Immunohistochemistry (IHC)

FFPE TMA specimens were heated at 60°C for 1 hour and subjected to a series of xylene and ethanol washes to remove paraffin. Antigen retrieval was performed with TRS buffer pH 9 (Dako) by heating in a microwave. The following primary antibodies were used in this study; Anti-FOXP3 mouse IgG1, Abcam cat# ab22510 at 17.0 μg/ml, Anti-CD4 rabbit IgG, Cell Marque cat# 104R-15 at 1.0 μg/ml, Anti-CD8 mouse IgG1 clone C8/144B, Dako cat# IS623, ready to use and Anti-PD-L1 rabbit IgG (E1L3N) cat # 13684, Cell Signaling Technology at 7.0 μg/ml. HRP system was used to detect signal. For all T cell markers tonsil tissue was used as a positive control and for PD-L1 placenta was the positive control.

### IHC scoring

**Fig 1:**
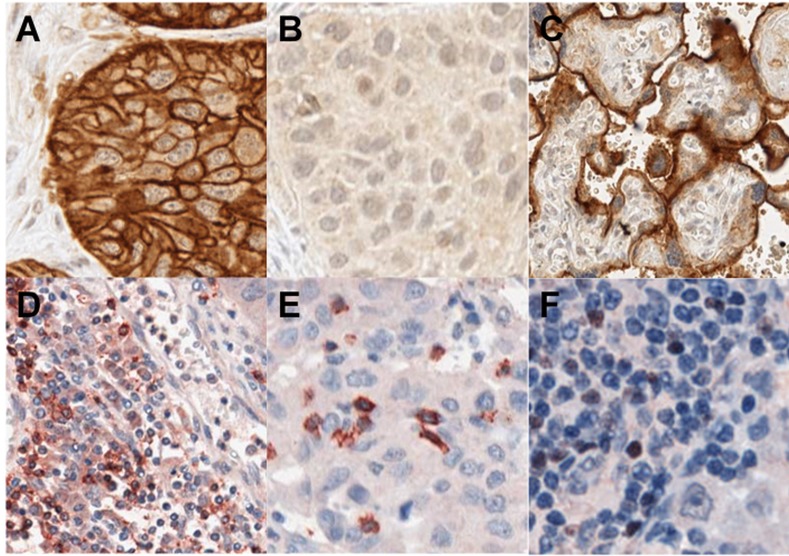
Representative images of immunohistochemical stains. (A) Strong PD-L1 staining. (B) Negative PD-L1 staining. (C) PD-L1 positive control. (D) CD8+ high stromal infiltrate. (E) CD8+ tumoral infiltrate. (F) FOXP3 positive stromal infiltrate.

After staining, slides were scanned using a high resolution scanner (ScanScope XT; Aperio) at 20x magnification. T cell markers were scored on a scale of 0–3 based on the percentage of positively stained cells, as a proportion of the total nucleated cells, in both the stromal peri-tumoral compartment and within the tumour itself, for the entire core (4 high powered fields) by one observer (MA), as described elsewhere.[8] A high stromal infiltrate was classified as having a stromal score of 3. FOXP3 lymphocytes were scored as above, and results, were dichotomized into high (score 2–3) and low (score 0–1) for analysis. PD-L1 was scored according to intensity of membranous staining (0–3) and the percentage of tumor cells stained, as reported elsewhere [30–33]. PD-L1 positivity was defined as >5% cells with membranous staining of intensity ≥ 2.[2, 31, 33, 34]. At the time of the commencement of the study, strong positivity was defined as of ≥ 50% cells with membranous staining of intensity ≥ 2, on the basis of results validating this cutoff for a different PD-L1 antibody clone.[2] Cutoff values used for this analysis were predefined. There was strong consistency between the triplicate cores for most cases, and in cases of discrepancy results were scored by the average between cores (for PD-L1 status, all cores were consistent in 397/420 (95%)). A subset of cases was observed by a pathologist (KA) independently (71 cases for CD8, 66 cases for FOXP3 and 82 cases for PD-L1), with discrepancies resolved by consensus. There was high concordance for FOXP3, CD8 and PD-L1 expression between scorers. For T cell markers and PD-L1, as results were dichotomized to high/low on the basis of scores, a linear weighted kappa was used to analyze inter-observer variability[35]. There was strong inter-observer reliability: the kappa values were 0.77, 0.79 and 0.83 for CD8, FOXP3 and PD-L1 respectively. Both scorers were blinded to the clinical outcomes of the patient. Due to low concordance and poor inter and intra-observer variability for CD4+ staining, these results have not been reported here. Although immune cells also stained for PD-L1 and were separately scored, there was significant variability in immune cell scores between the triplicate cores, and therefore these data have not been reported here. Representative images of IHC are shown in Fig 1.

### Mutational profiling

Gene mutation status was determined using Sequenom’s MassArray LungCarta panel as previously described. [36] All mutations were validated by Sanger sequencing.

### Statistical analysis

Survival analyses were performed to evaluate the association between marker expression and survival endpoints. Overall survival (OS) was determined from the date of surgery to the date of death. Disease-free survival (DFS) was determined from the date of surgery to the date of first relapse, and in cases of non-cancer related death, were censored at the time of death.

Marginal, stratified and by-group univariate analyses on OS and DFS were performed for CD8+ infiltrate, FOXP3 and PD-L1. P-values were computed using log-rank test, while hazard ratios (HR) and confidence intervals (CI) were derived from Cox’s model.

From univariate analyses, the following variables were chosen for the multivariate analysis: age, sex, pneumonectomy status, nodal stage, histology, smoking status, CD8+, FOXP3, and PD-L1. Interactions between CD8+, FOXP3, PD-L1 and nodal stage, histology and smoking were considered. Cox's proportional hazard model was used for OS and DFS separately. Variables were further selected by backward elimination based on Wald type test. The hierarchical structure of variables was maintained so that if interaction of a variable appears in the model, its main effect was preserved. After the backward selection procedure, proportional hazard assumptions were validated for each variable using Shoenfeld's test.

All analyses were performed on the R platform (version 3.1.3) using package *survival (version 2*.*38–3)*.

## Results

There were 527 patients in the entire cohort; 509 patients were assessable for CD8 status, 413 for FOXP3 status and 420 for PD-L1. Individual cores were excluded for analysis if there was significant necrosis or artefact. For the entire cohort, the median follow-up was 117 months and median overall survival was 46 months. Three hundred and forty-six patients were N0, 72 patients were N1 and 109 patients were N2. The median survival was 71, 32 and 14 months for N0, N1 and N2 disease respectively. As many of these cases occurred prior to 2004 (when the large adjuvant trials were initially reported) only 51 patients received chemotherapy (27 induction, 24 adjuvant). The median age was 67 and 51% of patients had died of disease recurrence. Smoking data were available for 507 patients. Smokers were classified as never smokers, light smokers (<25 packet years smoking) or heavy smokers (≥25 packet years smoking). There were 35 never smokers, 87 smokers and 385 heavy smokers. Most patients were Caucasian (96%).

Ninety-eight patients underwent pneumonectomy, with the remaining patients treated with lobectomy. *EGFR* mutations were found in 27 cases and *KRAS* mutations were detected in 100 cases. Other mutations (including *NRAS*, *TP53*, *BRAF*, *and PIK3CA)* were detected in 126 patients. Table 1 demonstrates clinico-pathological characteristics of the cohort and by PD-L1 status.

**Table 1:** Clinico-pathological characteristics of cohort.

*Characteristic*	*All patients (N = 527)*	*PD-L1 negative(N = 320)*	*PD-L1 strongly positive(N = 100)*	*p value(PD-L1 negative vs*. *PD-L1 strongly positive)*
**Sex n (% of total)**				
Male	365 (69.3)	220 (68.8)	77 (77)	0.11
Female	162 (30.7)	100 (31.3)	23 (23)	
**Median age**	67.2	68.0	63.6	0.04
**Nodal Stage n (%)**				
N0	346 (65.7)	204 (63.8)	65 (65)	0.13
N1	72 (13.6)	38 (11.9)	18 (18)	
N2	109 (20.7)	78 (24.4)	17 (17)	
**Histology n (%)**				
Adenocarcinoma	288 (54.6)	148 (46.3)	37 (37)	0.24
Squamous	182 (34.5)	109 (34.1)	38 (38)	
Other (mostly large cell)	57 (10.8)	63 (19.7)	25 (25)	
**Molecular n (%)**				
*EGFR* mutant	27 (5.1)	20 (6.3)	3 (3)	0.53
*KRAS* mutant	100 (19.0)	57 (17.8)	22 (22)	
Other mutation	126 (23.9)	75 (23.4)	24 (24)	
No mutation	274 (52.0)	168 (52.5)	51 (51)	
**Pneumonectomy n (%)**	98 (18.6)	65 (20.3)	16 (16)	0.39
**Smoking status n (%)**				
Heavy smoker	385 (73.2)	230 (71.9)	77 (77)	0.44
Light smoker	87 (16.5)	53 (16.6)	16 (16)	
Never smoker	35 (6.6)	24 (7.5)	3 (3)	
Unknown	20 (3.8)	13 (4.1)	4 (4)	

### PD-L1 staining

Utilizing a prespecified cutoff of 5% membranous staining, 43.6% of tumors were PD-L1 positive. Using a more stringent cutoff of 50%, 100 cases (23.8%) were classified as strongly positive (PD-L1+). Overall, apart from a tendency for slightly younger patients to be PD-L1+, there were no significant differences in strong PD-L1 expression by nodal status, pneumonectomy, smoking status, histology or molecular status (Table 1).

Only three of 27 never smokers had strong PD-L1 expression on their tumors. In smokers, strong PD-L1 expression was associated with a significantly improved OS (HR 0.75, 95% CI 0.57–0.99, p = 0.037) (S1 Table). There was a trend towards improved OS in strongly PD-L1 positive adenocarcinoma (HR 0.70, 95% CI 0.45–1.07, p = 0.096) compared to squamous cell carcinoma (HR 1.03, 95% CI 0.69–1.55, p = 0.885), although this was not significant. Strongly PD-L1 positive *EGFR* mutant NSCLC was associated with inferior prognosis (HR 7.05, 95% CI 1.62–30.61, p = 0.002), than *EGFR* wild-type NSCLC although these numbers were limited. *KRAS* status was not prognostic in the univariate or multivariate models. A test for interaction with CD8+ confirmed that strong PD-L1 status was not correlated with CD8+ expression.

In multivariate analysis PD-L1 expression was an independent predictor of improved OS and DFS (Table 2), in patients with N2 disease, although these data are based on 17 PD-L1+ and 109 PD-L1- patients. There were no significant differences in treatments received, with strong PD-L1 expression appearing to be prognostic across all N2 patients. Although the numbers were limited, nonetheless this does represent a large cohort of resected N2 patients. In contrast, there was a significant imbalance in known prognostic factors in the small group of patients with N1 disease. With 32% of PD-L1 negative patients having received adjuvant or neoadjuvant therapy, compared to 13% of strongly PD-L1 positive patients, this difference could account for the poorer survival observed in this subset. Moreover, 18% of PD-L1 negative patients had a pneumonectomy compared to 39% of strongly PD-L1 positive patients. Consequently, the disparate results seen in the small group of N1 patients may be due to imbalances in other, unaccounted for, prognostic factors that could not be included into the multivariate model.

**Table 2:** Multivariate analysis for OS, DFS.

	OS			DFS		
Characteristic	HR	95% CI	p-value	HR	95% CI	p-value
Age	1.03	[1.01, 1.04]	<0.001	*		
Pneumonectomy	1.67	[1.25, 2.24]	0.001	*		
N1 vs. N0	1.42	[0.95, 2.14]	0.088	1.58	[0.99, 2.51]	0.053
N2 vs. N0	3.68	[2.68, 5.04]	<0.001	3.94	[2.84, 5.46]	<0.001
CD8+	*			0.70	[0.50, 0.97]	0.034
PD-L1+ N0 vs PD-L1-	0.82	[0.57, 1.18]	0.282	0.76	[0.49, 1.17]	0.213
PD-L1+ N1 vs PD-L1-	1.88	[1.04, 3.41]	0.037	1.87	[0.95, 3.66]	0.068
PD-L1+ N2 vs PD-L1-	0.46	[0.26, 0.80]	0.006	0.49	[0.26, 0.94]	0.031

* Not utilized in final model after backward selection

### Stromal CD8+ tumor-infiltrating lymphocytes (TILs)

Overall, there were no significant differences in CD8+ expression by nodal status, pneumonectomy status, smoking status, histology or molecular status. Given that previous studies have shown prognostic differences associated with tumoral compared to stromal TIL, we further characterised the TIL using a similar score. Patients with a stromal CD8+ score of 3, without a corresponding high tumoral infiltrate were compared to tumors without a high stromal infiltrate. On stratified univariate analysis (S2 Table), there was an improvement in DFS in patients with a high stromal infiltrate (HR 0.72, 95% CI 0.54–0.97, p = 0.029) and a trend to significance in OS (HR 0.82, 95% CI 0.64–1.04, p = 0.096). The survival benefit was most evident in the N1 population where a significant benefit was observed in both OS (HR 0.44, 95% CI 0.21–0.90, p = 0.021) and DFS (HR 0.36, 95% CI 0.15–0.87, p = 0.017), albeit with the caveats mentioned above.

**Fig 2:**
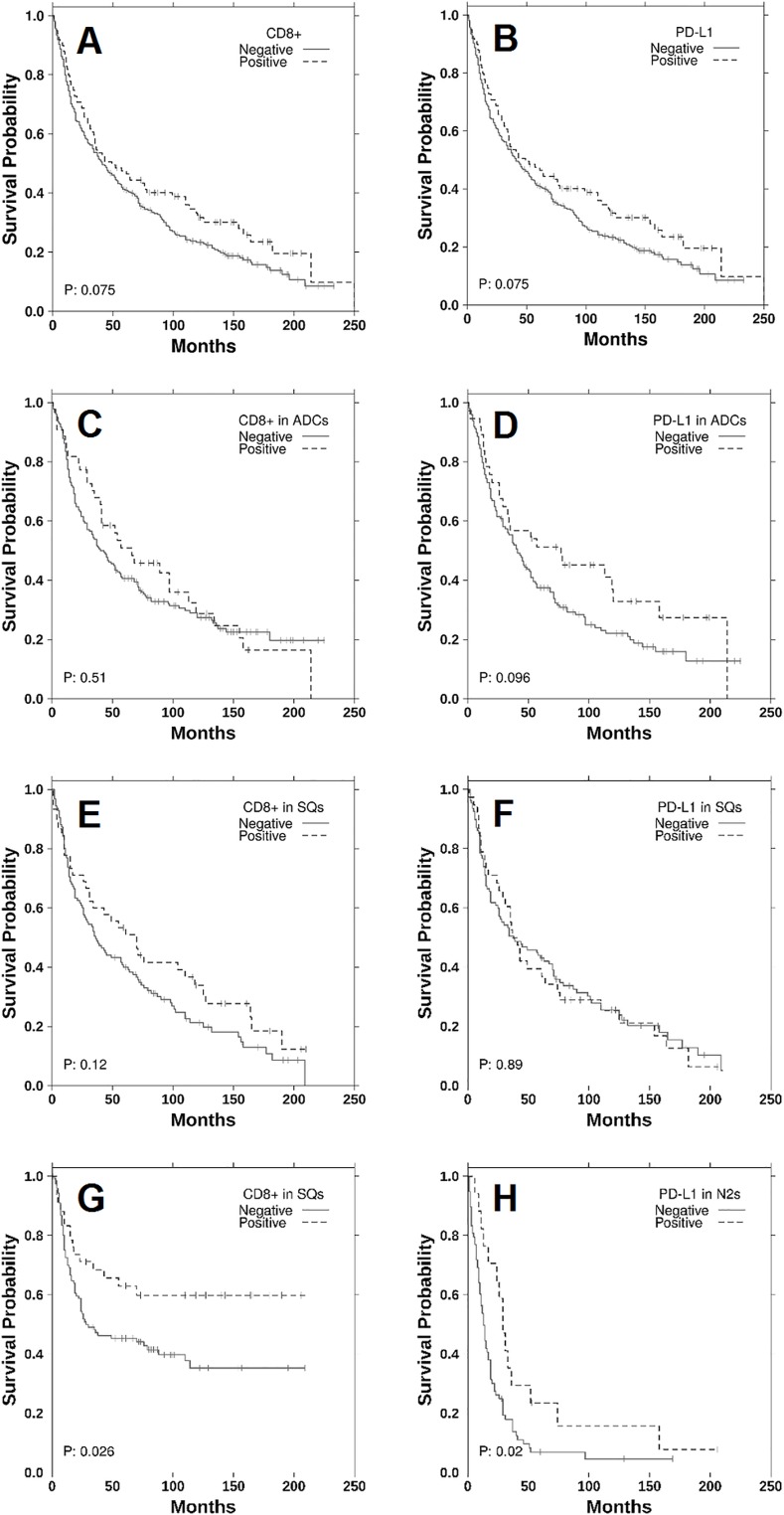
Kaplan-Meier curves of OS for CD8, PD-L1 status. (A) OS CD8+ TILs. (B) OS PD-L1. (C) OS CD8+ TILs in adenocarcinoma. (D) OS PD-L1 in adenocarcinoma. (E) OS CD8+ TILs in squamous cell cancer. (F) OS PD-L1 in squamous cell cancer. (G) DFS CD8+ TILs in squamous cell cancer. (H) OS PD-L1 in N2 disease.

There were no significant differences in OS between heavy, light and never smokers and CD8+ TILs. When stratified according to histology, high stromal CD8+ TILs was associated with significantly improved prognosis in squamous tumours (HR 0.55, 95% CI 0.32–0.94, p = 0.026) but not in adenocarcinomas (HR 0.83, 95% CI 0.54–1.28 p = 0.385). These findings were confirmed in multivariate analysis, with CD8+ expression being significantly associated with improved DFS but not OS (Table 2). The Kaplan-Meier survival curves according to PD-L1 and CD8+ expression are shown in Fig 2.

### FOXP3+ infiltrate

The FOXP3 score was dichotomized into FOXP3+ high (score of 2–3) and FOXP3+ low (score of 0–1) and analyzed for survival endpoints. In all patients, FOXP3 status was not associated with survival. In patients with N2 disease, there was a significantly improved DFS with high FOXP3 infiltrate (HR 0.59, 95% CI 0.35–0.99, p = 0.038), but no other associations were observed. FOXP3 positive status was strongly correlated with both increasing nodal stage and PD-L1 expression with significantly more strongly PD-L1 positive tumors also containing FOXP3 TILs (S3 Table).

### Matched nodal samples

In order to clarify whether PD-L1 status was maintained in nodal specimens, PD-L1 status was evaluated in a matched cohort of primary and mediastinal nodal samples from patients with N2 disease (n = 53). The concordance for reported PD-L1 status was 89%. The major cause of discordance was due to strong PD-L1 expression in the lymph node but absent or little expression within the primary sample in 4 (8%) samples.

## Discussion

Abrogation of the the PD-1/PD-L1 checkpoint using monoclonal antibodies is associated with not only improvement in response rates but also overall survival. Some predictive markers, such as *EGFR* mutations are both predictive but also favourably prognostic and it is important to determine the prognostic significance of PD-L1 in association with other immune cell infiltrates. These data demonstrate that tumoral PD-L1 expression was not prognostic in the overall cohort. Stromal CD8+ expression was prognostic in squamous cell histology but not adenocarcinoma. Although FOXP3 status was not independently prognostic, its expression was strongly correlated with advanced nodal stage and strong PD-L1 expression. Novel findings, include the strong concordance noted between primary tumors and their matched nodal primaries (in to date the largest published series of matched tumors) and a suggestion of poorer survival in never smokers with PD-L1 expression compared to smokers.

Strong tumoral expression of PD-L1 was demonstrated in 24% of cases, a finding consistent with the reported literature[37], with a commercially available assay which has been validated previously.[32, 38] Table 3 summarizes the literature published to date regarding PD-L1 in NSCLC. Although PD-L1 expression may correlate with responses to their inhibitors, the studies are plagued by a lack of uniformity in methodology of staining assessment, the antibody used and cutoff values for positivity.[37] Consequently, there is substantial debate regarding utilization of PD-L1 expression as a biomarker[37] and there are conflicting results regarding its prognostic influence in resected NSCLC.[4, 39–41] The two largest studies published to date [41, 42] suggest PD-L1 expression is associated with improved prognosis but have significant limitations. Velcheti et al[41] utilized a non-commercially available antibody, 5H1 and used an automated quantitative fluorescence method that is not widely reproducible. Cooper et al[42] did not pre-specify cutoff values to be used for PD-L1 positivity and reported a positivity rate of 8%, which is substantially lower than the published literature in NSCLC. Moreover, with PD-L1 status potentially reflecting poor prognosis disease in other epithelial malignancies[43], these inconsistent results raise significant queries regarding the role of the immune microenvironment in early stage disease, the answers to which may pave the way for more targeted studies of checkpoint inhibitors in the adjuvant setting.

**Table 3:** Reported studies of PD-L1 in NSCLC.

Author	Year	N	Population	PDL1 assay	PD-L1%	Cutoff %	Findings
Taube [31]	2014	41	Mixed histology	5H1, M3	53	5	Associated with TILsPredict response to chemotherapy
Azuma[44]	2014	164 NSCLC	Asian57% *EGFR* mutated Stage I-III	Lifespan Bio-sciences		Median	Associated with AC histology & *EGFR* mutation.
Konishi[40]	2004	52	Asian Stage I-III	M1H1		Median	No relationship to clinical outcomes observed
Kim[45]	2015	331 SCC	Asian Stage I-III	Cell Signalling	27	10	Correlated with CD8+ TILs Not associated with survivalCD8+ TILs correlated with improved survival
D’Incecco[33]	2015	125 NSCLC	Stage IVItalian	Abcam	55	5	Associated with AC histology, *EGFR* mutation.
Boland[46]	2013	214SCC	Caucasian Stage I-IV	Dako	19	1	No relationship to clinical outcomes observed.
Zhang[30]	2014	143 AC	Asian Stage I-III	Sigma-Aldrich		Median	Worse survival outcomes if PD-L1 positive
Velcheti[41]	2013	544 NSCLC	2 cohorts Caucasian & Greek Stage I-IV	Dr. Lieping Chen’s lab (5H1)	26, 35		Correlated with TILs Improved OS with PD-L1 expression (independent)
Yang[47]	2014	163 AC	Stage I Asian	Proteintech	40	5	Improved DFS PD-L1 positive No improved OS but very few deaths
Cooper[42]	2015	681 NSCLC	Caucasian Stage I-III	Merck	8	50	Improved OS PD-L1 positive in SCC, not in AC.
Schmidt	2015	321 NSCLC	Caucasian Stage I-III	Cell Signalling	24	5	Improved OS PD-L1 positive in SCC, adjuvant therapy, T2-T4 and N1-N3 disease.

AC adenocarcinoma; SCC squamous cell carcinoma; NSCLC non-small cell lung cancer; OS overall survival; DFS disease free survival; EGFR epidermal growth factor receptor; TILs tumor infiltrating lymphocytes.

The discrepant results in the N1 population of patients could not be explained by anomalies in the cohort, as standard prognostic factors such as age and nodal status were clearly prognostic in the multivariate model (Table 2). Rather, it is more likely due to the small number of strongly PD-L1 positive patients, which had a higher rate of pneumonectomy and lower overall use of adjuvant therapies (ie due to confounding factors). Conversely, in the N2 population, the observed effect was apparent irrespective of adjuvant therapy received. Of note, the use of adjuvant therapy was much higher in the N2 population (75%) than in the series as a whole (16%), possibly suggesting that the positive prognostic significance of PD-L1 in N2 disease reflects an interaction between PD-L1 expression and therapy. Overall, although these data suggest PD-L1 positivity may be favorably prognostic in N2 disease, the negative impact of PD-L1 in N1 disease and the lack of discernible prognostic effect in the N0 population which had less confounding, cast doubt over the strength of this effect.

Patients with strongly PD-L1+ *EGFR* mutant tumors appeared to have a markedly worse prognosis than wild-type patients, although caution must be taken when interpreting these data in light of the small number of never smokers and *EGFR* mutations. Interestingly, most studies reporting PD-L1 as a negative prognostic marker have come from Asian populations, where a high percentage of never smokers and *EGFR* mutations exist, whereas those reporting PD-L1 as a favourable prognostic marker have been predominantly in Caucasian smokers (Table 3). Taken together, this observation could reconcile some of the disparate results reported for PD-L1 and its impact upon prognosis in resected NSCLC, but would require further elucidation with a larger series of EGFR mutant tumors to make firm conclusions. There were too few ALK rearranged patients in this cohort to draw any meaningful conclusions from the data. *KRAS* status did not appear to independently influence prognosis or interact with PD-L1 or CD8+ status.

The high concordance of PD-L1 expression in matched mediastinal lymph nodes using immunohistochemical analyses in resected NSCLC has not previously been reported. As clinical decisions are increasingly made upon the presence of biomarkers, particularly in the setting of resource constraints, it was reassuring that samples from nodal specimens were concordant with the tumor primary.

TILs have long been thought to be prognostic in solid organ malignancies.[48] This has particularly been recognised in colorectal cancer, with a movement to develop a validated instrument, the immunoscore, to be added to standard prognostic markers.[49] Whilst some researchers have reported improved survival with CD8+ TIL infiltrates, [7, 8, 15] others have not.[23, 24] The most consistent data to date has been published by Donnem et al[8] which suggests a simple scoring scale for stromal CD8+ count can be highly prognostic. Our study used similar methodology and suggests that this is a reliable and reproducible methodology to add genuine prognostic information in a readily translatable fashion.

Our data suggest that PD-L1 expression may be favorably prognostic in locally advanced, resected NSCLC in a predominantly Caucasian population of smokers. Mechanistically, it remains unclear as to how PD-L1 expression might improve outcomes in resected disease. Given that PD-L1 is inducible, it is plausible that strong PD-L1 expression merely reflects a healthy homeostatic immune response to T cell activation, given the relative strength of TILs as a prognostic factor. However, this ignores the fact that there appears to be an independent association between PD-L1 expression and clinical outcomes on multivariate analysis.

There are several limitations to this study. The use of a TMA to analyze the immune infiltrate poses some limitations. TMAs represent only a small portion of tissue and could be subject to sampling error. To mitigate this, the use of triplicate cores from each patient somewhat diminishes this possibility. Additionally, the age of the tissue specimens may influence binding of the relevant antibodies to tissue, making the results difficult to generalize to a broader population. Moreover, TMAs are uniquely accessible to evaluate large series of patients’ such as ours in an efficient manner. Additionally, several series’ in a multitude of malignancies have now utilized IHC on TMAs for both PD-L1 and CD8+ expression.[8, 43] Finally, given the retrospective nature of the analysis and significant results being confined to subgroups, caution must be exercised over-interpreting the results.

As access to immune checkpoint inhibitors are likely to be linked to a PD-L1 assay, this large series of matched nodal specimens with primaries demonstrated reproducibility of PD-L1 expression between these sites and confidence that despite heterogeneity, there was good concordance. As immune checkpoint inhibitors move from the metastatic into the locally advanced and adjuvant setting, the prognostic role of their targets assumes greater importance, especially as these markers are inducible and much controversy exists regarding their predictive role in NSCLC.

## Supporting Information

S1 Table Univariate analysis by PD-L1 expression (PD-L1+ versus PD-L1-). (DOCX)

S2 Table Univariate analysis of prognostic significance of stromal CD8+ in NSCLC. (DOCX)

S3 Table FOXP3 and nodal status, PD-L1 expression. (DOCX)
