# Supplementary material for: PD-L1 and Tumor Infiltrating Lymphocytes as Prognostic Markers in Resected NSCLC
Source: PLoS One. 2016 Apr 22;11(4):e0153954. doi: 10.1371/journal.pone.0153954 (PMC4841565; doi:10.1371/journal.pone.0153954)
Supplement: S1 Table — (DOCX) [file pone.0153954.s001.docx]

**SUPPLEMENTARY APPENDIX**

***Table S1: Univariate analysis by PD-L1 expression (PD-L1+ versus PD-L1-)***

|  |  |  | **OS** | | | **DFS** | | |
| --- | --- | --- | --- | --- | --- | --- | --- | --- |
|  |  | N | HR | 95% CI | p | HR | 95% CI | p |
|  | **ALL** | 420 | 0.79 | [0.61, 1.03] | 0.075 | 0.79 | [0.58, 1.07] | 0.127 |
| Nodal | Stratified | 420 | 0.77 | [0.59, 1.00] | **0.047** | 0.78 | [0.57, 1.07] | 0.118 |
|  | N0 | 269 | 0.71 | [0.50, 1.01] | 0.057 | 0.72 | [0.47, 1.12] | 0.141 |
|  | N1 | 56 | 1.74 | [0.95, 3.18] | 0.065 | 1.82 | [0.92, 3.58] | 0.077 |
|  | N2 | 95 | 0.53 | [0.30, 0.92] | **0.02** | 0.51 | [0.27, 0.97] | **0.032** |
| Smoking | Stratified | 403 | 0.78 | [0.59, 1.03] | 0.073 | 0.79 | [0.57, 1.09] | 0.149 |
|  | Never | 27 | 4.34 | [1.14, 16.5] | **0.018** | 3.36 | [0.92, 12.28] | 0.052 |
|  | Light | 69 | 0.69 | [0.36, 1.33] | 0.267 | 0.55 | [0.23, 1.33] | 0.177 |
|  | Heavy | 307 | 0.76 | [0.56, 1.03] | 0.074 | 0.79 | [0.55, 1.13] | 0.186 |
|  | Stratified smokers* | 376 | 0.75 | [0.57, 0.99] | **0.037** | 0.75 | [0.54, 1.04] | 0.078 |
| Histology | Stratified | 420 | 0.80 | [0.61, 1.04] | 0.088 | 0.80 | [0.58, 1.09] | 0.149 |
|  | AC | 185 | 0.70 | [0.45, 1.07] | 0.096 | 0.72 | [0.44, 1.17] | 0.178 |
|  | SQ | 147 | 1.03 | [0.69, 1.55] | 0.885 | 1.01 | [0.61, 1.66] | 0.98 |
|  | Other | 88 | 0.63 | [0.35, 1.14] | 0.122 | 0.65 | [0.32, 1.31] | 0.224 |
| *EGFR* | Stratified | 182 | 0.67 | [0.43, 1.06] | 0.081 | 0.78 | [0.46, 1.30] | 0.335 |
|  | Wild | 159 | 0.59 | [0.36, 0.94] | **0.024** | 0.64 | [0.36, 1.12] | 0.109 |
|  | Mutant | 23 | 7.05 | [1.62, 30.6] | **0.002** | 9.73 | [2.10, 45.05] | **0.000** |
| *KRAS* | Stratified | 237 | 0.63 | [0.44, 0.91] | **0.013** | 0.60 | [0.38, 0.93] | **0.020** |
|  | Wild | 158 | 0.59 | [0.37, 0.94] | **0.025** | 0.64 | [0.37, 0.93] | 0.115 |
|  | Mutant | 79 | 0.71 | [0.40, 1.27] | 0.245 | 0.53 | [0.25, 1.10] | 0.078 |
