# Supplementary material for: PD-L1 and Tumor Infiltrating Lymphocytes as Prognostic Markers in Resected NSCLC
Source: PLoS One. 2016 Apr 22;11(4):e0153954. doi: 10.1371/journal.pone.0153954 (PMC4841565; doi:10.1371/journal.pone.0153954)
Supplement: S2 Table — (DOCX) [file pone.0153954.s002.docx]

***Table S2: Univariate analysis of prognostic significance of stromal CD8+ in NSCLC***

|  |  |  | **OS** | | | **DFS** | | |
| --- | --- | --- | --- | --- | --- | --- | --- | --- |
|  |  | N | HR | 95% CI | p | HR | 95% CI | p |
|  | **ALL** | 509 | 0.82 | [0.64, 1.04] | 0.096 | 0.72 | [0.54, 0.97] | **0.029** |
| Nodal | Stratified | 509 | 0.86 | [0.67, 1.09] | 0.205 | 0.75 | [0.56, 1.01] | 0.055 |
|  | N0 | 335 | 0.95 | [0.71, 1.28] | 0.748 | 0.84 | [0.57, 1.22] | 0.360 |
|  | N1 | 68 | 0.44 | [0.21, 0.90] | **0.021** | 0.36 | [0.15, 0.87] | **0.017** |
|  | N2 | 106 | 0.95 | [0.57, 1.60] | 0.846 | 0.88 | [0.50, 1.55] | 0.658 |
| Smoking | Stratified | 491 | 0.82 | [0.64, 1.05] | 0.107 | 0.71 | [0.53, 0.96] | **0.026** |
|  | Never | 34 | 0.57 | [0.13, 2.42] | 0.435 | 0.71 | [0.16, 3.09] | 0.644 |
|  | Light | 84 | 1.20 | [0.68, 2.13] | 0.531 | 0.67 | [0.31, 1.43] | 0.290 |
|  | Heavy | 373 | 0.77 | [0.58, 1.01] | 0.059 | 0.72 | [0.52, 1.01] | 0.056 |
| Histology | Stratified | 509 | 0.80 | [0.63, 1.02] | 0.073 | 0.74 | [0.55, 1.00] | **0.044** |
|  | AC | 226 | 0.88 | [0.60, 1.29] | 0.508 | 0.83 | [0.54, 1.28] | 0.385 |
|  | SQ | 173 | 0.74 | [0.50, 1.09] | 0.119 | 0.55 | [0.32, 0.94] | **0.026** |
|  | Other | 110 | 0.80 | [0.47, 1.34] | 0.388 | 0.92 | [0.51, 1.66] | 0.774 |
