# Supplementary material for: PD-L1 and Tumor Infiltrating Lymphocytes as Prognostic Markers in Resected NSCLC
Source: PLoS One. 2016 Apr 22;11(4):e0153954. doi: 10.1371/journal.pone.0153954 (PMC4841565; doi:10.1371/journal.pone.0153954)
Supplement: S3 Table — (DOCX) [file pone.0153954.s003.docx]

***Table S3: FOXP3 and nodal status, PD-L1 expression***

| **Characteristic** | | **FOXP3 score** | | **p-value**  **Fisher test** |
| --- | --- | --- | --- | --- |
|  |  | Negative | Positive |  |
| Nodal | 0 | 116 | 150 | 0.001 |
|  | 1 | 14 | 44 |  |
|  | 2 | 24 | 65 |  |
| PD-L1 status | Negative | 107 | 166 | 0.021 |
|  | Positive | 22 | 65 |  |
